# Supplementary material for: In Vitro/In Vivo Translation of Synergistic Combination of MDM2 and MEK Inhibitors in Melanoma Using PBPK/PD Modelling: Part I
Source: Int J Mol Sci. 2022 Oct 26;23(21):12984. doi: 10.3390/ijms232112984 (PMC9656205; doi:10.3390/ijms232112984)
Supplement: Supplementary file 1 [file ijms-23-12984-s001.zip › ijms-1990351-supplementary.pdf]

Supplementary Materials

In Vitro/In Vivo Translation of Synergistic Combination of MDM2 and MEK Inhibitors in Melanoma Using PBPK/PD Modelling: Part I

Jakub Witkowski, Sebastian Polak, Zbigniew Rogulski and Dariusz Pawelec

Table S1. Siremadlin and Trametinib resistant population estimates. For MTS assay $n = 4$, last observation after 72h and for RealTime-Glo assay $n = 3$ and last observation after 80h.

Compound	Assay	Max concentration (nM)	% of viability at the last measurement ± SD
Siremadlin (HDM201)	MTS	200	21.910 ± 5.922
	RealTime-Glo	4000	20.980 ± 6.855
	Mean	-	21.445 ± 6.389
Trametinib	MTS	4	13.340 ± 2.596
	RealTime-Glo	40	14.854 ± 2.592
	Mean	-	14.097 ± 2.594

Table S2. Drug interaction between Siremadlin and Trametinib on A375 cells. For RealTime-Glo assay $n = 3$. Synergyfinder package analysis.

Timepoint [h]	*n*	Synergy ZIP δ	Synergy Loewe δ	Synergy HSA δ	Synergy Bliss δ	Models score δ
12	3	9.293	2.319	-1.391	9.931	5.038
24	3	11.459	-3.098	5.120	14.929	7.103
28	2	5.976	1.196	7.997	8.925	6.023
32	2	4.878	2.821	8.816	6.720	5.808
36	3	5.396	2.149	11.654	7.441	6.660
48	3	6.083	4.289	14.661	6.339	7.843
52	3	2.903	7.124	13.463	3.046	6.634
56	3	3.548	8.466	14.243	3.455	7.428
60	3	3.967	9.633	14.754	3.859	8.054
72	3	5.505	10.613	16.316	5.432	9.467
76	3	5.069	12.106	16.498	4.989	9.666
80	3	5.252	12.737	16.733	5.199	9.980
Mean (datapoints >24h)		4.858	7.113	13.513	5.540	7.756
SD		1.346	4.355	3.111	1.957	1.614

Table S3. Drug interaction between Siremadlin and Trametinib on A375 cells. For RealTime-Glo assay *n* = 3. Synergy package analysis.

Timepoint (h)	*n*	beta	alpha12	alpha21	gamma12	gamma21	R^2
12	3	0.194	1.150	0.703	63.546	18.590	0.299
24	3	0.656	0.888	2979.928	7.420	0.691	0.734
28	2	0.318	1.418	74,999.607	0.742	68,773.056	0.886
32	2	0.334	1.688	50,000.132	1.156	0.052	0.940
36	3	0.280	1.071	65.504	1.040	0.565	0.938
48	3	0.244	1.547	1.746	1.032	0.543	0.977
52	3	0.227	1.636	1.123	0.871	0.497	0.988
56	3	0.218	1.757	1.484	0.865	0.592	0.990
60	3	0.193	2.583	1.583	0.914	0.697	0.989
72	3	0.224	3.012	1.363	0.837	0.578	0.989
76	3	0.210	3.162	1.359	0.744	0.667	0.992
80	3	0.194	3.074	1.453	0.805	0.680	0.992
Mean (t >24h, R^2>0.8)		0.244	2.095	12,507.535	0.901	6877.793	0.968
SD		0.050	0.780	26,999.124	0.136	21,747.779	0.036
Mean (t >48h, R^2>0.8)		0.216	2.396	1.444	0.867	0.608	0.988
SD		0.018	0.727	0.195	0.091	0.075	0.005

Table S4. Synergyfinder analysis parameters.

Synergyfinder parameter	Selected option	Notes
Data type	Viability	-
Impute	True	Option to handle missing values in drug combination matrices if there were any
Noise	False	There was no addition of small random numbers to response data
Correction	Non	There was no adjusting of the baseline of the response matrix

Table S5. Synergy analysis parameters.

Synergy parameter	Selected option/range	Notes
MuSyC variant	full	-
E0 bounds	(0.8, 1.2)	-
E1 bounds	Automatically selected bounds based on round min/max E1 values	rounded to 1 decimal place
E2 bounds	Automatically selected bounds based on round min/max E2 values	rounded to 1 decimal place
E3 bounds	Automatically selected bounds based on round min/max E3 values	rounded to 1 decimal place
Bootstrap iterations	100	-
Max_nfev	5000	maximum number of function evaluations, a high number to ensure that model will converge

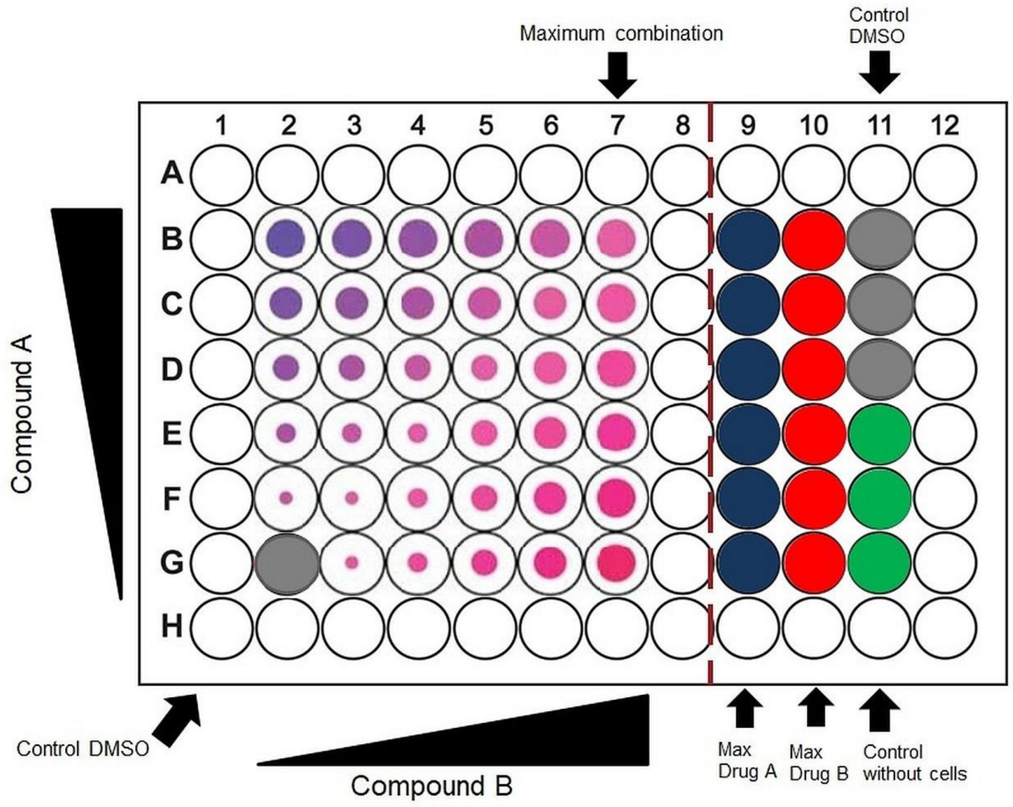

Figure S1. MTS assay plate layout used in combination study. Compound A (Drug A – represents HDM201). Compound B (Drug B – represents Trametinib). Control without cells consisted of an appropriate volume of cell culture medium.

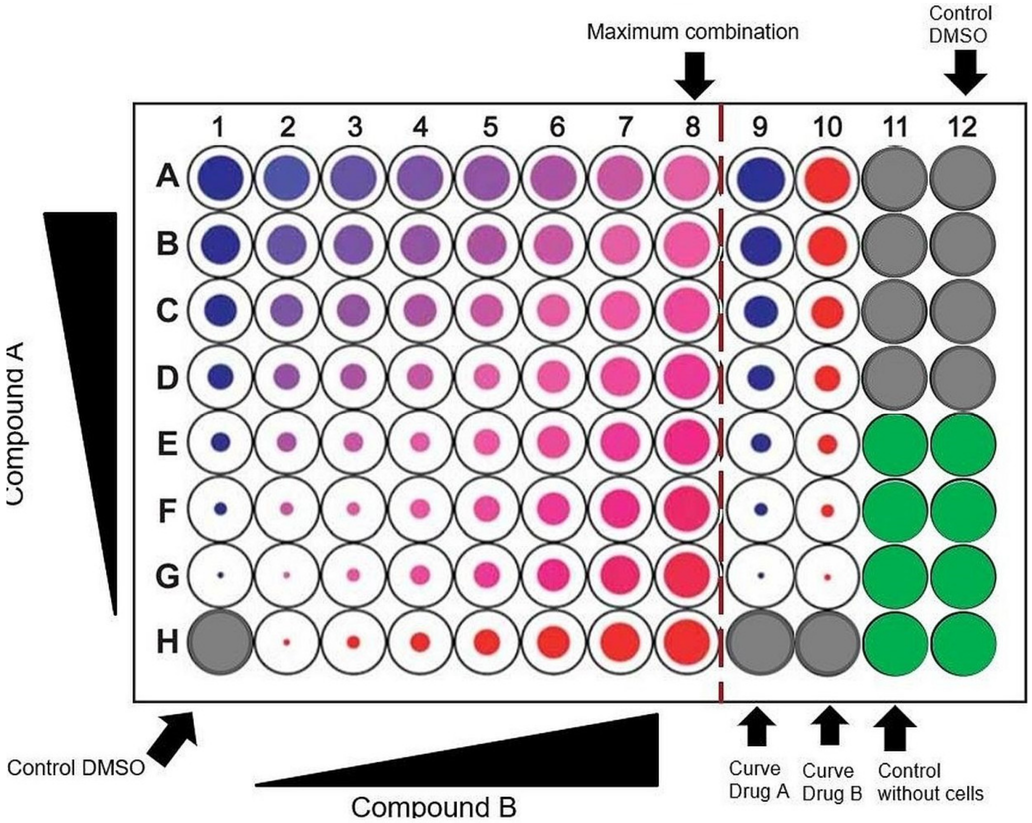

Figure S2. RealTime-Glo assay plate layout used in combination study. Compound A (Drug A – represents HDM201). Compound B (Drug B – represents Trametinib). Control without cells consisted of an appropriate volume of cell culture medium.

Code S1. R script for drug combination analysis with the use of Synergyfinder package. The script is processing all files in the input directory and prints drug interaction metrics in the console and log.

```
# R code for calculating drug interaction metrics using Synergyfinder package.

# load needed libraries
# futile.logger library provides a logging utility in order to follow the analysis progress

library(synergyfinder)
library(readxl)
library(xlsx)
library("futile.logger")

# set your input and output directories
currentPath = getwd()
inputDirPath = file.path("set here your input path")
outputDirPath = file.path("set here your output path")

options(width = 60)

# read your input data excel file
prepareInputData <- function(file) {
  input_file = file.path(inputDirPath, file)
  input_data <- read_excel(
    input_file,
    col_types = c(
      "numeric",
      "text",
      "text",
      "numeric",
```

```
            "numeric",
            "numeric",
            "text",
            "text"
          )
        )
        # set Synergyfinder analysis parameters
        data <- ReshapeData(
          input_data,
          data.type = "viability",
          impute = TRUE,
          noise = FALSE,
          correction = "non"
        )
        return(data)
      }
      # preparing your output catalog structure based on assumption that particular experiments in input data will have following structured name: Exp (N) (cell line name) (drug 1 name)+(drug 2 name)
      prepareExperimentDir <- function(file) {
        setwd(outputDirPath)

        # directory_name = sub("Exp \\d\\s", "", file)
        directory_name = strsplit(file, " ")[[1]][3]
        dir.create(directory_name, showWarnings = FALSE)

        localDirPath = file.path(outputDirPath, directory_name)

        setwd(localDirPath)

        return(localDirPath)
      }
      # calculating drug interaction metrics using particular models (methods)
      countScores <- function(data) {
        scoreZIP <- CalculateSynergy(data = data, method = "ZIP")
        attr(scoreZIP, "method_name") <- "ZIP"
        scoreHSA <- CalculateSynergy(data = data, method = "HSA")
        attr(scoreHSA, "method_name") <- "HSA"
        scoreBliss <- CalculateSynergy(data = data, method = "Bliss")
        attr(scoreBliss, "method_name") <- "Bliss"
        scoreLoewe <- CalculateSynergy(data = data, method = "Loewe")
        attr(scoreLoewe, "method_name") <- "Loewe"

        scores = list(scoreZIP, scoreHSA, scoreBliss, scoreLoewe)

        return(scores)
      }
      # Saving plots showing drug interaction patterns at different concentrations calculated with particular methods
      SaveScoreFiles <- function(scores, file, localDirPath) {

        summary_scores <- list()

        for (score in scores) {
          plot_filename_2d = paste("synergy", attr(score, "method_name"), file, "2D", sep = " ")
          # plot synergy method returns summary score values
          summary_score = PlotSynergy(
            score, "2D",
            save.file = TRUE,
            file.type = 'jpeg',
            file.name = c(plot_filename_2d)
          )
          #add calculated metrics method name (ZIP/HSA/Bliss/Loewe)
```

```
        summary_scores[attr(score, "method_name")] <- summary_score

      }
      return(summary_scores)
    }


    # operations during processing particular files
    ProcessExperiment <- function(file) {
      data = prepareInputData(file)

      #adjust file name for synergyfinder images
      file = strsplit(file, "[.]")[[1]][1]

      localDirPath = prepareExperimentDir(file)

      scores = countScores(data)

      summary_scores = SaveScoreFiles(scores, file, localDirPath)
      #printing summary scores for particular methods (ZIP/HSA/Bliss/Loewe) in console and log
      flog.info(paste("ZIP: ", summary_scores[1]))
      flog.info(paste("HSA: ", summary_scores[2]))
      flog.info(paste("Bliss: ", summary_scores[3]))
      flog.info(paste("Loewe: ", summary_scores[4]))

      setwd(currentPath)
    }


    ####################################################################
    # ----- SETTING THE MAIN LOOP - process all files in the input path ----#
    ####################################################################

    # list all files in input path
    all_files = list.files(path = inputDirPath)
    # setting the logging utility
    flog.appender(appender.tee('logs.txt'))
    flog.info("----- WORK STARTED -----")

    for (file in all_files) {
      tryCatch({
        flog.info(paste("Going to process file: ", file))

        ProcessExperiment(file)

        flog.info(paste("Finished processing file: ", file))
      }, error = function(err) {
        setwd(currentPath)

        flog.error(paste("Error occured while processing file: ", file))
        flog.error(err)
      })
    }

flog.info("----- WORK DONE -----")
```

Code S2. Python script for drug combination analysis with the use of Synergy package. The script is processing all files in the input directory and prints drug interaction metrics in the console and log.

```
# Python code for calculating drug interaction metrics using Synergy package.

# load needed libraries
import pandas as pd
import numpy as np
import os
import sys
import logging
import openpyxl
from pathlib import Path
# import models which will be used to calculate drug interaction metrics
from synergy.combination import MuSyC

def process_synergy(src_file):
    # add information about actually processed file in console and log
    logging.info("Processing file %s" % src_file)
    print("Processing file %s" % src_file)
   # read your input data excel file and setting the dataframe (read data from first sheet)
    df = pd.read_excel(src_file, sheet_name=0)
    df['drug1.conc'] = df['drug1.conc']
    df['drug2.conc'] = df['drug2.conc']
    d1 = df['drug1.conc']
    d2 = df['drug2.conc']
    d1_name = df['drug1_name'][0]
    d2_name = df['drug2_name'][0]
    cell_line_name = df['cell_line_name'][0]
    experiment_number = df['experiment_number'][0]
    effect = df['effect']

   # automatic bounds values selection based on rounded min/max values for control, drug 1, drug 2 and their combination wells
    # indicating indexes for control, drug 1, drug 2 and their combination from which bounds will be calculated
    E0_indexes = [0]
    E1_indexes = [6, 12, 18, 24, 30]
    E2_indexes = slice(1, 6)
    E3_indexes = [slice(7,12), slice(13,18), slice(19,24), slice(25,30), slice(31,36)]

    # get values for particular bounds
    E1_values = effect.array[E1_indexes]
    E2_values = effect.array[E2_indexes]
    E3_values = []
    for group in E3_indexes:
        E3_values = np.append(E3_values, effect[group].array)

   # setting bounds values
    E0_bounds = (0.8, 1.2)
    E1_bounds = (round(min(E1_values), 1), round(max(E1_values), 1))
    E2_bounds = (round(min(E2_values), 1), round(max(E2_values), 1))
    E3_bounds = (round(min(E3_values), 1), round(max(E3_values), 1))

   # printing selected bounds in console and log
    logging.info("E0 bounds: %s - %s" % E0_bounds)
    logging.info("E1 bounds: %s - %s" % E1_bounds)
    logging.info("E2 bounds: %s - %s" % E2_bounds)
    logging.info("E3 bounds: %s - %s" % E3_bounds)
    print("E0 bounds: %s - %s" % E0_bounds)
    print("E1 bounds: %s - %s" % E1_bounds)
    print("E2 bounds: %s - %s" % E2_bounds)
```

```
            print("E3 bounds: %s - %s" % E3_bounds)

        # setting bounds for MuSyC model
            musyc_model_full = MuSyC(E0_bounds=E0_bounds, E1_bounds=E1_bounds, E2_bounds=E2_bounds,
E3_bounds=E3_bounds, variant="full")

            # calculating drug interaction metrics using MuSyC model
            musyc_model_full.fit(d1, d2, effect, bootstrap_iterations=100, max_nfev=5000)

        # printing calculated drug interaction metrics and fit goodness in console and log
            # logging.info(musyc_model_full.summary())
            logging.info("AIC - %s" % musyc_model_full.aic)
            logging.info("BIC - %s" % musyc_model_full.bic)
            logging.info("R^2 - %s" % musyc_model_full.r_squared)
            print("AIC - %s" % musyc_model_full.aic)
            print("BIC - %s" % musyc_model_full.bic)
            print("R^2 - %s" % musyc_model_full.r_squared)
            parameters = musyc_model_full.get_parameters(confidence_interval=95)
            logging.info(parameters)
            print(parameters)

    # set your input and logs directories
    input_dir_path = "set here your input path"
    logs_dir_path = "set here your logs path"

    # prepare logs
    if not os.path.exists(logs_dir_path):
        os.mkdir(logs_dir_path)

    # logger setup
    logging.basicConfig(level=logging.INFO, filename='log/main.log', format='%(asctime)s :: %(levelname)s ::
%(message)s')

    ### MAIN LOOP ###
    with os.scandir(input_dir_path) as entries: # iterate over files in input directory
        for entry in entries:
            # take only xlsx files to process
            if entry.is_file() and (entry.name.endswith('.xlsx') or entry.name.endswith('.xls')):
                try: # process each file
                    process_synergy(os.path.join(input_dir_path, entry.name))
                except Exception as err: # log info about errors
                    logging.info("ERROR IN FILE %s --- %s" % (entry.name, err))

        logging.info('Done.')
```
